# Acceptability of Using a Decision Aid to Support Family Carers of People With Dementia Towards the End of Life: A Qualitative Study

**DOI:** 10.1111/hex.14123

**Published:** 2024-06-19

**Authors:** Nathan Davies, Narin Aker, Emily West, Greta Rait, Elizabeth L. Sampson

**Affiliations:** ^1^ Research Department of Primary Care and Population Health, Centre for Ageing Population Studies University College London London UK; ^2^ Marie Curie Palliative Care Research Department, Division of Psychiatry University College London London UK; ^3^ PRIMENT Clinical Trials Unit, Research Department of Primary Care and Population Health University College London London UK; ^4^ Department of Psychological Medicine, Royal London Hospital East London NHS Foundation Trust London UK

**Keywords:** decision‐aid, decision making, dementia, palliative care, qualitative

## Abstract

**Objectives:**

To explore the experiences, acceptability and utility of a decision aid for family carers of people with dementia towards the end of life.

**Methods:**

We conducted semi‐structured interviews with a sample of family carers enroled into a 6‐month feasibility study in England, sampling to gain a range of experiences and views, based on relationship to person they cared for (e.g., spouse, adult child), age, gender, and self‐reported use of the decision aid during the feasibility study. Interviews were conducted in March 2021–July 2021 and analysed using reflexive thematic analysis. We used COREQ checklist to report our methods and results.

**Results:**

Family carers found the decision aid acceptable, describing it as comprehensive, accessible with relevant information and its presentation enabled good engagement. Experiences of the decision aid covered four main themes which demonstrated the perceived acceptability and utility: 1. A source of support and reassurance; 2. Empowering conversations and confidence; 3. Including the person living with dementia; and 4. Breaking down complexity.

**Conclusions:**

An aid focussing on decisions about dementia end of life care supported family carers break down complex and emotive decisions, not only with making decisions in the moment but also in future planning.

**Patient or Public Contribution:**

Our three Patient and Public Involvement (PPI) members (all former family carers) were crucial throughout the wider study. PPI supported development of the topic guides, supported trialling the topic guide and interview procedures and finally supported the development of themes as part of the analysis.

**Abbreviations**
CFIR: The Consolidated Framework for Implementation Research
COREQ: Consolidated Criteria for Reporting Qualitative Research
ODSF: Ottawa Decision Support Framework
PPI: Patient and Public Involvement

## 1 Introduction

People with dementia are faced with a variety of decisions, including decisions about healthcare, lifestyle, finance, legal, as well as decisions about everyday aspects of their lives [1, 2, 3]. However, as an individual's dementia progresses, their ability to make their own decisions deteriorates [4]. When a person no longer has the capacity to make their own decisions, the Mental Capacity Act in England and Wales states decisions should be made in their best interests [5]. An individual may have made an advance directive (formerly known as living wills) which inform decisions to be made, however in practice these are rare. Family carers may have a Lasting Power of Attorney for Health and Welfare (LPA) to make decisions on behalf of the individual when they no longer have capacity. Decisions should be made together with the health care professional in charge of the individual's care. However, family carers often feel solely responsible for making these decisions [1] and professionals rely on family carers to be able to report the wishes and preferences of the individual [6]. Best practice recommends that decisions should be shared between professionals, family carers and, where possible, with the person with dementia themselves to ensure person‐centred care [7, 8].

Previous studies have demonstrated that family carers find making decisions about their relative's care difficult, and in particular, decisions around end‐of‐life care [9]. This often leads to feelings of guilt, anxiety, mistrust and confusion [9]. Many studies report that support for family carers of people with dementia would be beneficial [1, 9, 10]. Multiple frameworks have been developed to encourage support for decision making in healthcare [11, 12]. The Ottawa Decision Support Framework (ODSF), which aims to support decision making across a broad range of decisions, states that those making decisions have a series of decisional needs including knowledge and expectations, which affects the outcome of decisions. Outcomes include the quality of the decision and the impact of the decision. The ODSF demonstrates that this relationship can be mediated by decision support, such as decision aids [11].

Decision aids are tools which can support patients with decisions, explicitly stating the decision to be made, providing information about the decisions and options available, including associated benefits and harms [13]. There is substantial evidence demonstrating the effectiveness and feasibility of decision aids to support decision making among patients and family carers including improving patient knowledge and expectations, across a range of advanced and chronic conditions [13, 14]. Similarly, decision aids have been found to increase knowledge and reduce decisional conflict at the end of life among older adults [15] and people with dementia [15].

As part of a larger study, we co‐produced with people with dementia, family carers, and professionals, a decision aid for family carers supporting people with dementia towards the end of life [16]. Previous decision aids in dementia care have focussed on one specific decision each, for example moving into long‐term care or topics such as goals of care which may cover multiple decisions [17, 18]. However, for those with dementia towards the end of life, decisions are unlikely to be isolated, but rather challenging and complex situations that necessitate several decisions for family carers and professionals in a short period of time. There is therefore a need to develop and test a decision aid which better reflects this reality and encompasses several decisions.

As part of a 6‐month feasibility study, this paper reports the qualitative evaluation which aimed to explore the experiences, acceptability and utility of a decision aid for family carers of people with dementia towards the end of life. Specific objectives were to explore:

1. Their experiences, acceptability and utility of the use of the decision aid;
2. Barriers and facilitators to using the decision aid;
3. Recommendations for further refinement of the decision aid for future use and larger scale evaluation.

## 2 Methods

### 2.1 Design

A qualitative study using semi‐structured interviews in England March 2021–July 2021, nested within a 6‐month feasibility study. The study has been reported using the consolidated criteria for reporting qualitative research (COREQ) checklist [19]. The quantitative results of the feasibility study are published separately [20].

### 2.2 Participants and Recruitment

All family carers (*n* = 20) who took part in the feasibility study were invited to take part in an interview. Twelve participants accepted the invite to an interview, with 10 interviewed after 2 later declined to be interviewed. We felt the 10 who accepted the invite and were interviewed was enough to provide in‐depth and meaningful information guided by the principles of information power [21]. Using the concept of information power we considered the quality of the interview data, the descriptive aim of our study and our analysis strategy to decide on the number of interviews.

### 2.3 Procedure

As part of the feasibility study participants used the decision aid for 6 months, with follow up points at three and 6 months to collect quantitative outcome measures. At the 6‐month follow‐up appointment all participants were invited to take part in a qualitative interview and provided with a participant information sheet and consent form. Those who were interested provided written, electronic, or verbal consent. Interviews took place via telephone or using Microsoft Teams. Nine interviews were completed by NA a female research assistant trained in qualitative research methods and who had also collected quantitative data with the participants, and one interview by ND a male senior qualitative researcher with a psychology background. Interviews were guided by an interview schedule which was developed and piloted with our Patient and Public Involvement (PPI) group, consisting of three former family carers. The schedule included questions on why participants chose to take part in the study, how they used the decision aid, who they used the decision aid with, barriers and facilitators to using the decision aid, content, and the delivery of the decision aid (see appendices). We used the Consolidated Framework for Implementation Research (CFIR) to inform questions, to ensure we covered all relevant information about the intervention's acceptability, feasibility, and barriers/facilitators to use [22]. The CFIR has been used in similar decision aid studies [23]. CIFR consists of five domains (1) intervention characteristics, (2) the outer setting (i.e., contexts such as covid), (3) inner setting (i.e. family dynamics and relationships with professionals), (4) characteristics of individual participants, and (5) process. Interviews were recorded using either an encrypted Dictaphone or Microsoft Teams.

### 2.4 Analysis

Interviews were analysed using reflexive thematic analysis [24, 25]. Interview audio files were transcribed verbatim and checked against the audio file for accuracy. ND and NA read through all transcripts to familiarise themselves with the data and made notes on the transcripts of key and interesting ideas in relation to the aim of the study. NA created a combination of inductive and deductive codes through familiarisation and applied these to two transcripts. These coded transcripts were sent to ND and EW and all three authors met to discuss the coding, adding additional codes, removed duplicate codes and defined codes. ND coded all remaining interviews with ongoing discussions with NA, adding and refining codes, using a combination of inductive and deductive approaches. Through several meetings all authors met to discuss the development of themes from the codes, themes were refined, defined, and agreed by all authors. This team approach to analysis allowed for the input of different interpretations and ideas. The analysis team consisted of two researchers from psychology backgrounds, one from medical anthropology, a GP, and a psychiatrist. Themes were presented to our PPI group, for sense checking, further feedback, and refinement. All data was managed using NVivo 12 [26].

### 2.5 Ethics

London Queen Square Ethics Committee and Heath Research Authority approved the study in March 2020 (18/LO/0408).

### 2.6 Decision Aid

The developed decision aid is an interactive paper‐based booklet which covers: (1) changes in care, (2) eating and drinking difficulties; (3) everyday wellbeing for person with dementia; and (4) healthcare, tests and medication. The decision aid includes information about options available, opportunities for family carers to reflect on their own values and preferences, as well those of the individual, useful resources and contacts, myth busters, and top tips. In using the decision aid, the family carer is then able to record a preference and use the decision aid as a tool to discuss with the health care professionals and others involved in the decision‐making process. The decision aid content was co‐produced with people with dementia, family carers and professionals, using data from a review and interviews with people with dementia, family carers and professionals [1, 16, 17, 27, 28, 29, 30]. Full details about the content and co‐production of the decision have been published elsewhere [16].

## 3 Results

Ten participants were interviewed (see Table 1), with interviews lasting approximately 60 min each. Overall participants suggested the decision aid was acceptable, and it was positively received. Participants discussed their experiences and views on acceptability and utility across four main themes: (1) A source of support and reassurance; (2) empowering conversations and confidence; (3) including the person living with dementia; and (4) breaking down complexity.

**Table 1:** Participant demographics and demographics of the people with dementia they care for.

Demographic information, results are displayed as *n* (%), unless specified otherwise	Person with dementia being cared for by participants, *N* = 10	Carer, *N* = 10
Age, mean (SD)	85.7 (9.4)	67.1 (8.5)
Gender, female	6 (60)	9 (90)
Ethnicity		
White	10 (100)	10 (100)
Residence		
Owner‐occupied	—	9 (90)
Housing association rented	—	1 (10)
Private home, no health services	3 (30)	—
Private home, with social services	3 (30)	—
Residential home	1 (10)	—
Other nursing home	2 (20)	—
Other (sheltered accommodation)	1 (10)	—
First language		
English	10 (100)	10 (100)
Highest level of education		
No qualifications	3 (30)	—
O levels	1 (10)	1 (10)
A levels (or post O level)	2 (20)	—
Degree	2 (20)	5 (50)
Postgraduate	2 (20)	4 (40)
Religion		
Christian	8 (80)	4 (40)
No specific	2 (20)	6 (60)
Lives with		
Alone	3 (30)	
Spouse	3 (30)	
Other	1 (10)	
N/A (i.e., live in a care home)	3 (30)	
Relationship to person with dementia		
Spouse		4 (40)
Child		4 (40)
Sibling		1 (10)
Other		1 (10)
Dementia type		
Alzheimer's Disease	5 (50)	
Frontotemporal dementia	1 (10)	
Vascular dementia	2 (20)	
Dementia with Lewy bodies	1 (10)	
Mixed dementias	1 (10)	

### 3.1 A Source of Support and Reassurance

The decision aid provided a source of support for participants with managing care, making decisions and the managing the emotional consequences of being a family carer. The decision aid helped them feel mentally prepared to make decisions.

Participants discussed the difficulties of being a family carer and a decision maker on behalf of someone who was no longer able to care for themselves. The decision aid helped family carers consider and manage their emotional wellbeing which had been impacted by caring and the burden of making decisions, ‘it makes me feel better about myself’ (017). They discussed how the aid was a comprehensive yet succinct resource to support them:

It was useful too—it was the first time I'd had something comprehensive that I could refer to.

(042)

The decision aid evoked a variety of different emotions, both positive and negative which acted to both facilitate but also discouraged the use. Some of the decisions were emotionally challenging and at times overwhelming. For some these reinforced acts of denial and led to them ‘block[ing] it out’ (042):

[The topic] changes in care, I guess I could work through, because that's something that was relevant to me, but it is emotionally challenging because that change of care is, by its very nature, challenging and emotionally challenging.

(038)

Despite the challenge of the topics and emotions evoked by the decision aid, for many they were already experiencing these emotions before, and the decision aid provided an emotional support. It provided them strength and a framework to reflect on what was best or needed, confront acts of denial and challenge feelings of guilt. The decision aid provided a source of reassurance, for some relief and reaffirmed the decisions they had made and reduced the feeling of being overwhelmed. In many circumstances the decision aid ultimately helped and motivated them to act:

It helped me not to feel guilty that I wanted to get somebody else to come and give me time to go out.

(017)

But having that [decision aid] gives you confidence you're making the right choices. Choices maybe you don't want to make but they are the right ones. And after making those decisions, it's like a weight lifted, a relief. Whereas I thought I'd feel really awful and guilty and all the rest of it. No, I didn't, I felt, ‘yeah, OK, that was right’. (042)

Being able to manage their emotions helped to feel mentally prepared, and more informed despite the unpredictable nature of dementia which many acknowledged:

[…] it's given me a sort of long‐range view of what I might be expected to be doing and what other possibilities there are in coping with it [dementia and being a family carer], which I didn't really have before. Because I found the whole format was really well‐structured and taking me through various stages of things to think about and what might happen in the future. So I think that I have benefited in that I feel I've got a more stable idea of what I'm doing, really, if that's any help.

(017)

I am going to use it next week when I meet the end of life planning people. […] we are very soon I think [going to] have to start looking for funding and a placement [care home]. Because of the deterioration in the last month. And so I can say I've got the criteria. I've read through this and you know, and he meets this and he meets that. So I just feel I'm prepared and ready for when I meet them.

(033)

It was also an opportunity for some to reflect on their role, and what it meant to be a family carer. This helped them to realise and understand the impact of this role on themselves, and their wellbeing. Overall, the decision aid reduced the feeling of being alone and increased the feeling of being supported through feeling informed, prepared, and reassured.

### 3.2 Empowering Conversations and Confidence

The decision aid worked as a communication tool for many, empowering them to have conversations and the confidence to discuss issues such as eating difficulties and other topics covered in the decision aid.

Some discussed how the decision aid had empowered discussions between family carers, people with dementia, other family members and professionals, providing not just knowledge but also the confidence to have discussions and ask informed questions:

We had a meeting with her GP some time ago and he was very impressed with what I was saying. He said, ‘You seem to know more about this than I do’. So, I feel confident that I'm aware of the situation in a realistic way, that anything that needs to be done will be done at the appropriate time. Which is actually laid out in the booklet [decision aid].

(020)

When you first sent it [decision aid] to us and I shared it with my husband, [it was] a catalyst for us to talk about that [end of life care] and about potentially any decisions we might make.

(032)

The decision aid was a tool to discuss with family, decisions which needed to be made. It helped manage disagreement among families and was used by some as a way of negotiating with their other family carers about care for the individual. For example, in the quote below a daughter caring for her mum used the decision aid to explain to her brother why a balanced diet was not the most important consideration at that stage, but it was more important for their mum to eat what she would like to have regardless of nutritional value:

He [brother] would say increasingly […], having too much of that [food] is bad for someone. And I could actually say, ‘Look, you're wrong. Look’.

(022)

There were mixed experiences of using the decision aid with professionals. Many had not used it with them and explained they had not seen anyone to use it with. Some participants had used it as a reference point, with one family carer explaining their GP photocopied what she had written in there for their records:

They photocopied some of my comments and things. Because she [GP] said that would help, you know, when I contact them.

(033)

### 3.3 Including the Person Living With Dementia

Within interviews we explored if participants used the decision aid with people with dementia, or to consider what the person themselves would want, considering their values. None of the participants interviewed managed to engage the person with dementia with the use of the decision aid. Many of the participants acknowledged that they did not try sharing it with the person with dementia. As the participants were caring for someone with dementia towards the end of life, many individuals were too advanced to work with the decision aid, they did not feel sharing the decision aid with the person themselves would be helpful and some suggested the person would be uncomfortable viewing it. Family carers often felt the need to protect the person with dementia; however, this could result in the exclusion of the individual from discussions about their own care:

I wouldn't share it with my dad, that's all, just because he just wouldn't understand and he doesn't like to—if I try and involve him in any discussions, he just tends to sort of shut down in a way.

(034)

The decision aid does not provide support or tips on engaging a person with dementia, nor was it designed for use by people with dementia. Future developments should look at how to actively engage a person with dementia to ensure meaningful shared decision making for example, alternative formats (video, pictorial):

Yeah [did share it with person with dementia], but he didn't really understand. He looked at the pictures […] And to me his dementia is so advanced he didn't really… I mean, I could say he looked at the pictures and he did want to turn one page to see what was on the next page, but he doesn't retain anything anyway, so it's not like I wasn't involving him. So, I mean he didn't express any opinions about it, he is nearly non‐verbal so it's really hard to get anything from him.

(033)

Despite this, the decision aid did encourage family carers to reflect on the values and wishes of the person with dementia, which is an alternative way of including the individual in decision making when they may no longer be able to actively participate. Many discussed how they knew what the person would want, or they had previously discussed preferences, but the decision aid helped to bring that consideration to the forefront of their minds:

I've always tried to do [name]'s wishes because I know them and so if you always try to do that, that's really what the decision aid is like.

(008)

I think it's [decision aid] just made me think about the important thing, that dad feels happy. There's certain things that he just won't do, or there's no point making him do something just because I think it's going to be good for him. So, if he doesn't want to do it and he's happy enough as he is, just that acceptance really.

(034)

### 3.4 Breaking Down Complexity

For many family carers decision making can be seen as overwhelming and all consuming. A key aim of the decision aid was to break down the decisions so they were less overwhelming and the complexity was reduced. This was achieved through careful design of the contents and features of the decision. Participants explained the content within the decision aid and the way in which it was presented/formatted made the decision making process more manageable:

In general, it's helping me move towards not being so overwhelmed by decisions.

(038)

Information was presented in a way which was easy to understand and broken down into understandable bites instead of large swathes of information, as one participants said ‘some of the stuff, with the best will, it is a bit dense’ (060) and difficult to work through. The decision aid provided almost a framework for making decisions:

It's a kind of a structure [the decision aid] […]. There are questions that can be asked outside the commercial decision, the principles or the concept, this is this type of care we're looking for or this is why we're looking for this type of care, or this is how we're going to arrive at a decision. I think that's structure is really useful.

(038)

To break down complexity it was important the decision aid was engaging. This was achieved not only by what information was included but also how it was presented, including language which was easy to comprehend:

It's not sort of vague and airy‐fairy, it's all pretty well cut and dried. Which is what you need because otherwise things get a bit sort of up in the air and things don't get done properly as you want them to be done.

(020)

Participants liked the variety of features, and the way information was presented making it more accessible and encouraged use, including the use of illustrations which again helped to break down dense pieces of text and information. Other features included frequently asked questions, myth busters, and fictional scenarios, where participants were able to consider how the scenario related to their experience, prompting them to think through:

I thought the frequently asked questions were good. I like the fact that it mixed up short, sharp little bits and then more contentious things.

(022)

## 4 Discussion

### 4.1 Summary

The positive views from family carers who used the decision aid in this study indicates that it is feasible and acceptable for a decision aid to include more than one decision. This is important as it reflects the reality of caring for someone with dementia towards the end of life, where multiple intertwined decisions may need to be made in a short period of time.

The positive feedback on the decision aid compliments previous work with decision aids that has demonstrated positive effects in improving expectations for the future and increasing knowledge [13, 14], and the on‐going call for the development of decision support tools to help this population [31]. This is particularly important as many families are often unaware of what may happen in the later stages of dementia or find that engaging in discussions about the later stages can be difficult. The decision aid provided a source of useful information for family carers, although some acknowledged this information would have been better provided earlier in the dementia trajectory when they had experienced greater uncertainty.

However, it is not simply a lack of information that makes decision making complex and difficult. As participants discussed, some decisions can feel overwhelming and previous studies have demonstrated decisions can be emotionally challenging [31]. Making decisions about end of life care can lead to feelings of guilt, which directly influences the decisions that are made [32]. Providing support through a decision aid for such decisions broke down the complexity, providing a clear framework and supporting family carers to think through decisions, reflecting not just on their own values but those of the person with dementia too, an important aspect of shared decision making and proxy‐based decision making [1, 14, 28]. The decision aid may help to contain or take on some of the emotional burden and guilt.

Participants did not use the decision aid much with professionals; however, this study took place during the initial stages of the COVID‐19 pandemic in England when there was less availability of services and family carers were uncertain about engaging support and help [33, 34]. However, in cases where the decision aid was used with professionals, it allowed family carers to feel prepared and informed, which participants reported was commented upon by professionals. The decision aid may enable family carers to prepare in advance what questions to ask, and what information and support they would like to receive from health and social care teams before their appointments. This may be a way to empower family carers and support them to feel confident in their caregiving.

Similarly, participants did not use the decision aid with people with dementia, explaining that the person with dementia would no longer be able to comprehend and process the information. Some felt the decision aid should be available earlier, as this would ensure a person living with dementia themselves could make use of the aid, providing opportunity. This would align with the principles of shared decision making to ensure the person living with dementia is included in decisions about their care and the basic principles of the Mental Capacity Act that all people should be supported to make their own decision where possible [5]. Reviews have demonstrated the ability of people with dementia to remain involved in the decision making process [35, 36], and it is important people with dementia remain involved in their care and in making decisions [37]. Introducing the decision aid earlier when the person with dementia has capacity could help engage the individual in conversations and be useful for planning for future care. Previous work has demonstrated that people with dementia often lack information and understanding about the physical changes in the later stages of dementia, for example about the potential development of eating and drinking difficulties [29]. This decision aid may help improve knowledge among people with dementia, as family carers have reported, but also act as a way to engage in discussions with them about future care. This study suggests the use of alternative formats may improve engagement with people with dementia for example, the use of graphics to aid discussions, or the use of videos which has been used in previous decision aids [38, 39].

### 4.2 Strengths and Limitations

Although a small sample of ten participants, this reflects nearly half of the participants who completed the 6‐month feasibility study. Inclusion of participants who were lost to follow up would have strengthened the interview data. This study was undertaken during COVID‐19 when family carers were more stressed and had greater difficulties with caring, which may have influenced the experience of the decision aid and interview responses. Participants in this study were all white and predominantly Christian, which is not reflective of the greater population in England and limits transferability of findings to other ethnicities, cultures and religious groups.

### 4.3 Implications for Research, Policy and Practice

This positive evaluation by participants suggests a larger scale evaluation of the decision aid should be conducted to understand its effectiveness in supporting decisions and explore in more depth its acceptability. It is important that this work considers future implementation, including implementation strategies and models of delivery to maximise reach and impact. It is particularly important to look at where in the health and social care systems the decision aid may be introduced and by who to maximise reach and uptake. Many of the lessons learnt from this study are applicable to development of decision aids in general, especially in dementia and older adults and not just this specific decision aid that was tested.

In future iterations of the decision aid consideration should be given to address accessibility and low health literacy with additional formats of the decision aid such as audio or video delivery. Many participants were supportive of a paper version, however some participants expressed a desire for alternative formats such as online and an app‐based version. The COVID‐19 pandemic has accelerated digital healthcare [40, 41], and it is important interventions such as the decision aid keep pace with these developments.

Alternative formats, such as audio and video, may increase engagement and accessibility for people with dementia. It is important to pay particular attention to how people with dementia can be included in decision making throughout the dementia trajectory, however this is complex and requires in‐depth exploration therefore was out of scope for the current study which focussed on family carers. This may lead to the inclusion of top tips on how to include someone with dementia or alternative versions of existing sections.

Further work is needed to test the decision aid with populations from different ethnicities, cultures and religions, taking note of modifications needed to fit with what matters to different groups of individuals.

Although comprehensive, no decision aid will ever cover all the topics of importance to provide high‐quality care. Other topics which should be explored include dental, eye care and hearing loss in people with dementia. We are aware of ongoing studies which are exploring decision making within some of these fields.

## 5 Conclusions

It is important to evaluate and explore the implementation of the decision aid for family carers. Future use should consider providing the decision aid to those in the earlier stages of the dementia trajectory, including people with dementia. There is scope for the development of further decisions aids in other areas of dementia care using the lessons learnt from this study.

## Author Contributions

**Nathan Davies:** conceptualisation, investigation, funding acquisition, writing–original draft, methodology, writing–review and editing, formal analysis, supervision, data curation. **Narin Aker:** writing–review and editing, formal analysis, data curation. **Emily West:** supervision, formal analysis, writing–review and editing. **Greta Rait:** writing–review and editing, funding acquisition. **Elizabeth L. Sampson:** writing–review and editing, funding acquisition, formal analysis.

## Ethics Statement

London Queen Square Ethics Committee and Heath Research Authority approved the study March 2020 (18/LO/0408).

## Consent

We collected informed written or recorded verbal consent. All the steps/methods were performed in accordance with the relevant guidelines and regulations.

## Conflicts of Interest

The authors declare no conflicts of interest.

## Supporting information

Supporting information

## Data Availability

The data that support the findings of this study are available on request from the corresponding author. The data are not publicly available due to privacy or ethical restrictions.
