# Supplementary material for: Acceptability of Using a Decision Aid to Support Family Carers of People With Dementia Towards the End of Life: A Qualitative Study
Source: Health Expect. 2024 Jun 19;27(3):e14123. doi: 10.1111/hex.14123 (PMC11187896; doi:10.1111/hex.14123)
Supplement: Supplementary file 1 — Supporting information [file HEX-27-e14123-s001.docx]

**APPENDIX 1: INTERVIEW TOPIC GUIDE**

**PART A: EXPERIENCE OF BEING A PARTICIPANT**

1. What interested you in this study and the decision aid?
   1. What were your expectations?
   2. Did you think it would help you with decisions or care for the person?
2. What has been your experience of being a participant? (EXPLORE WHY)
   1. Time commitment to complete assessments/interviews
   2. How did you find the questionnaires? Any particularly challenging too long etc.
   3. Research materials/research team support
   4. Shaped your experience of caring and moving forward
   5. Explore remote experience because of covid-19.

**PART B: EXPERIENCE OF USING DECISION AID**

1. Generally, how and when have you been using the decision aid?
   1. Using it to plan or at times when you need to use it?
   2. Overall experience of using it (pros cons etc, why)
2. Can you give me an example of when you have used it?
   1. How did you use it in this situation?
   2. Who did you involve in the decision?
   3. Did you discuss or use the decision aid with anyone else for this example?
   4. Why did you use the decision aid for this example?

***Use the below questions with the example they provide to qu 4.***

1. Have you used the decision aid with your healthcare team?
   1. What were their views of it?
   2. How did this impact on the way you use it? (i.e. how did their support alter the way you use it?/encourage you to use it or not)
   3. Have you shown it to or spoken to your GP or other professionals about it?
      1. If so, what was your experience of this?
   4. Have you taken it to any medical appointments?
   5. Was it a useful resource to use with them?
   6. Has the decision aid helped or altered communication with them?
2. Have you used the decision aid with your family or friends?
   1. What were their views of it?
   2. How did this impact on the way you use it? (i.e. how did their support alter the way you use it?/encourage you to use it or not)
   3. How would you have involved these people before?
   4. Was it a useful resource to use with them?
   5. Has the decision aid helped or altered communication with them?
3. What about using it with the PLWD themselves?
   1. how did you engage with them?
   2. What were the reasons you used it with them or did not?
   3. Did this help? Consider structuring discussions prompting or encouraging discussions.
   4. Would you have involved them before?
   5. Was it a useful resource to use with them?
   6. Has the decision aid helped or altered communication with them?
4. Is there anyone you did not deliberately share it with?
   1. Why not?
5. How do you think the decision aid has influenced how you make decisions?
   1. Views/preferences
   2. Values of yourself or what you think the pwd would value
   3. Has it affected your confidence or feeling of self-competence?
   4. Changes to knowledge?
   5. Goals of care for the person
6. What has the study led you to do or change?
   1. Catalyst for further conversations/decisions/ action?
   2. Did this lead to more support?
   3. Feel prepared?

**PART C: CONTENT AND FORMAT**

1. Is there anything not in the decision aid which you think should be?
   1. (e.g. legal issues such as POA, financial issues such as benefits, care home guidance)
   2. Particular decisions
   3. More options
   4. More information about benefits and disadvantages
2. What were the most helpful aspects?
   1. Other people’s stories and experiences
   2. Benefits and disadvantages
   3. Information
   4. Reflective pieces
   5. Support networks
3. How do you think we could make the decision aid more workable for someone like yourself?
   1. Technology, app, online, lined to care records?
4. Did you have comments on the design and the format of the decision aid?
   1. Wording
   2. Complexity
   3. Use of images and cartoons – simplistic
   4. Tables and bullet points
5. What should our priorities be for the decision aid next?
   1. Make changes – develop it further
   2. Make available to others
   3. Stop using it
   4. Different format – make available digitally?
   5. Make available at an earlier stage of dementia and the PLWD
   6. Make available to charities and NHS services

**PART D: BARRIERS AND FACILITATORS**

1. What do you think are the main things that may have stopped you from using the decision aid?
2. How has using the decision aid made you feel?
   1. What has been the emotional impact?
   2. Are some parts more challenging to discuss than others? Why?
3. Have there been other things going on that have got in the way?
   1. Illness; Family dynamics or challenges
   2. How has COVID-19 impacted your using the decision aid and being involved in the study?
4. What do you think encouraged you to use the decision aid?

**PART E: DELIVERY**

1. In this study you received the decision aid through the post and the researcher discussed it with you if you wanted. Was this enough?
   1. Some suggest a decision coach to support you is helpful – what do you think? What would that look like?
   2. In the future if you were to be giving this how would you want to receive it?
   3. Sent it or just giving it to look at privately
   4. Someone sitting down with you explaining the aspects
   5. Someone sitting down with you and helping you complete all aspects
   6. Who would these people be? Explore clinical people, trust in person, authority of person.
2. When is the right time to introduce this decision aid?
   1. Is it helpful to plan or when needing to make the decision?

**PART F: SUMMARY**

1. What would you tell others about the decision aid?

***Close***

- Check if the participant would like to add anything not already covered
- Summarise some take home messages from the interview
- Thank the participant for their time today and taking part in the whole study
- Ask the participant to complete a demographic checklist and provide gift voucher.
